# Primary healthcare implementation in practice: Evidence from primary healthcare managers in Ghana

**DOI:** 10.4102/phcfm.v12i1.2183

**Published:** 2020-05-20

**Authors:** Nana Nimo Appiah-Agyekum

**Affiliations:** 1 Department of Public Administration and Health Services Management, University of Ghana Business School, Legon, Accra-Ghana

**Keywords:** primary healthcare, Ghana, Ghana health service, implementation, healthcare

## Abstract

**Background:**

Primary healthcare (PHC) is a core part of healthcare in developing countries. However, the implementation of PHC since its inception in developing countries has been lethargic, inconsistent and marred by controversies.

**Aim:**

This study investigates some of the controversies surrounding PHC implementation. It also examines how PHC is being implemented in Ghana as well as how the approaches adopted by PHC implementers influence PHC outcomes in developing countries.

**Setting:**

This study is set in Ghana and involves national, regional and district managers of PHC.

**Methods:**

A qualitative case study was used to gather information from 19 frontline PHC managers through semi-structured interviews. Interviews were recorded and transcribed. They were then qualitatively analysed using the thematic framework analyses approach.

**Results:**

Findings uncover a lack of clear meaning of what PHC is and how it should be approached amongst key implementers. It also shows discrepancies between official policy documents and directives, and actual PHC practices. Findings also show a gradual shift from Alma Ata’s comprehensive PHC towards a more selective and intervention-specific PHC. Whilst donor and external stakeholders’ influence are the key determinants of PHC policy implementation, their support for vertical and other medicine-based interventions have gradually medicalised PHC.

**Conclusion:**

There is a need to pay more attention to understanding and addressing the gaps in PHC implementation and its inconsistencies. Furthermore, the role and control of donors and external development partners in PHC policy formulation and implementation, and their concomitant effects on community participation and empowerment, must be critically examined.

## Introduction and problem analysis

Primary healthcare (PHC) is ‘essential care based on practical, scientifically sound and socially acceptable methods and technology, made universally accessible to individuals and families in the community through their full participation, and at a cost that the community and country can afford to maintain at every stage of their development in the spirit of self-reliance and self-determination’.^1^ Owing to the comprehensive and wide-reaching implications of this definition, PHC to date remains a nebulous concept with several issues arising from the context, components and feasibility of the approach.^2,3^

Principally, the definition above was constructed around the public health failings in developing countries^4,5^ and the expression of the desired pathway for achieving health outcomes similar to that of developed countries. Consequently, Akin et al.^6^ describe the PHC movement as merely an international effort to expand and redirect health service programmes in developing countries. This may be because the conditions that necessitated the summit as well as the content of the definition limit its relevance and applicability to developing countries. Primary healthcare has therefore received its greatest support and application in developing countries, where it operates as a standalone national health programme.^7,8^

Within this context, the primary novelty of PHC was its demand for a universally accessible essential care programme to address inequalities in healthcare access and outcomes across different sections of the population.^9,10^ This was necessary because governments had prioritised capital investments and training medical personnel at the expense of expanding access.^11^ Consequently, PHC’s glorified maxim of ‘Health for All’ was more than a slogan. It was a denunciation of the ‘health for some’ or ‘health for a few’ that existed in many parts of the developing world^12^ and a call for increased and sustained access to health. Access to health in the developing world is as relevant today was it was in the pre-PHC era with millions still lacking full healthcare or any care at all in spite of the significant investments made in PHC.^13,14^ Improving access therefore calls for a re-examination of PHC implementation, especially in developing countries where PHC has found its widest support, yet socio-economic and locational barriers to access are rife.^15,16^

Generally, the essence of PHC implementation in developing countries cannot be overemphasised. This is in view of the fact that the outcomes of PHC initiatives vary widely as a result of the differing levels of commitment, acceptance and resource investments for implementation.^17,18^ In addition, Widmar^19^ suggests that intervention-specific and contextual factors born out of the meanings attached to PHC influence the extent and success of PHC and in effect the outcomes of its implementation. Similarly, McPake and Mensah^20^ link the success or otherwise of PHC and its associated programmes to issues revolving around implementers, implementation systems and approaches to implementation.

Yet, a summative review of the existing information on PHC uncover a paucity of empirical studies on the approaches to PHC implementation. Even the limited studies available have focussed on particular interventions (for instance Christopher et al.^21^) and professional groups (for instance Crisp^22^) rather than providing a holistic outlook of the key issues influencing its implementation. Whilst data from frontliners of PHC initiatives are essential and have been used in several studies on PHC, there is still a dearth of evidence from the top- and middle-level managers of PHC implementation who drive the process.

This study therefore addresses the gaps identified above by providing first-hand evidence from middle- and top-level implementers on key issues surrounding how PHC implementation is approached. Using Ghana as an example, it discusses how the approaches adopted by PHC implementers influence PHC outcomes in developing countries and further addresses the paucity of information on the subject.

## Methodology

The methods of this study were guided by El Bindari-Hammad and Smith’s^23^ guide to PHC implementation assessment and the policy implementation assessment tool by Bhuyan et al.^24^ Drawing strength from the above tools, this study adopted the qualitative case study approach. The qualitative case study approach was suitable because it facilitates the analyses of persons, events, decisions, periods, projects, policies, institutions or other systems in a holistic fashion.^25^

Based on McPake^9^ findings that PHC implementation in developing countries is driven more by top- and middle-level implementers than by lower-level actors, this study focussed on key personnel involved in managing and implementing PHC activities across Ghana. Sampling was therefore purposive and used Yin^26^ analytical sampling concept to identify and select key informants within a defined context based on the analytical merits of the experiences they share rather than their quantitative representativeness.

The sampling frame in this study was limited to Ghana Health Service (GHS) officers in charge of PHC implementation at the national, regional and district levels. Besides having an in-depth knowledge on PHC, these persons were better positioned to provide anecdotes and practical evidences by virtue of their active roles in translating, directing, evaluating and managing PHC implementation. Respondents were sampled through a combination of convenience and snowballing sampling procedures. These procedures were considered ideal for the study in view of the practical challenges of making contact, gaining access and getting information from the top-level persons in GHS across the 10 regions and 216 Metropolitan Municipal and District Assemblies in Ghana. Patton^27^ and Berg and Lune^28^ also lent support to their use in studies where prospective respondents are not easily accessible or to navigate the barriers imposed by administrative red-tapism. As recommended by Bhuyan et al.,^24^ sampling involved enlisting the aid of a national officer, in this case the deputy director general of the GHS, who then helped recruit respondents at the regional levels. Regional-level officers were then engaged to help recruit respondents at the district levels. Sampling was ended at the 19th respondent after saturation was reached (Table 1).

**TABLE 1:** Summary table of sample.

Level	Designation	Number
National	Deputy director general, GHS	1
	Director, health promotion division	1
Regional	Regional director of GHS	3
	Deputy regional director of GHS	3
	Regional health research officer of GHS	3
District	Metropolitan, municipal and district director of health	8
**Total**		**19**

Interviews were used as the main data collection instrument based on their ease of administration and proven efficacy in other similar studies. The interviews were semi-structured and aided by an interview guide. The interview guide was not restrictive and designed based on the study objectives, themes and issues gathered from a preliminary literature review. It was also guided by El Bindari-Hammad and Smith^23^ PHC assessment tool. Interviews were held at the offices of the respondents and lasted between 60 min and 142 min. Interviews were recorded, transcribed and later signed-off by respondents prior to analysis.

Ethical approval for the study was obtained from the Noguchi Memorial Institute for Medical Research Institutional Review Board (NMIMR-IRB). Issues reviewed by the IRB covered informed consent processes, compensation, anonymity, confidentiality and full disclosure to study participants. Implications and utilisation of research findings, possible risks, discomforts and rights of participants were also reviewed. This study was thus covered by ethical clearance certificate numbers NMIMR-IRB CPN 087/12-14. This study was also reviewed by the GHS, which then granted permission to involve its personnel, facilities and programmes in the study.

The data collected were analysed qualitatively using Ritchie and Spencer^29^ thematic framework analysis. Framework analysis is a variant of thematic analysis, which was developed specifically for applied policy research, thus making it suitable for use in this study. After signing off the transcribed data, analysis was done by sequentially following the framework analysis steps put forward by Pope et al.^30^ Analysis thus began by immersion into the raw data, followed by the development of thematic frameworks and then indexing. Charting was then followed finally by mapping and interpretation. It is at the final stage that charts were created to define concepts, map the range and nature of phenomena, create typologies and find associations between themes with a view to providing explanations for the findings. The analysis was then discussed within the context of the reflexivity exercise and relevant literature.

### Ethical consideration

Ethical clearance was obtained from the Noguchi Memorial Institute for Medical Research on 04 July 2013 (clearance number: NMIMR-IRB CPN 087/12-14).

## Key findings

Several meanings of PHC were constructed by interviewees although they worked within similar organisational environments. The various meanings provided however emphasised the medicalised nature of PHC in Ghana, with respondents associating PHC with the lowest level of medical services and professionals and hospital-based care:

‘Refers to the lowest level of medical care often provided in rural and deprived communities.’ (R3, Male, District Director of Health Services)

Respondents were also knowledgeable of the key approaches, actors and interventions that embodied PHC implementation in Ghana with constant reference being made to Community-based Health Planning and Services (CHPS) as the main vehicle for PHC implementation in Ghana. However, consensus was that, because of the multiplicity of stakeholders, interventions and approaches, PHC implementation in Ghana was but a constant attempt to synergise several vertical, stand-alone and monolithic approaches and programmes:

‘… [*W*]e are always struggling to balance the interests of external PHC stakeholder … several stakeholders supporting various initiatives under CHPS in Ghana.’ (R7, Male, Regional Director of Health Services)

Findings also suggest that the approaches for managing PHC implementation were driven more by donor preference and priorities than by the Ghanaian government, Ministry of Health (MoH) and GHS. Consequently, the approaches used to implement PHC had been lethargic, poorly controlled, lacked continuity and focus, and often without expected impacts:

‘PHC initiatives are mostly donor interventions so it’s approached according to donor wishes …. everything is subject to their approval and we have no control over how PHC is approached … we are just cogs in a wheel!’ (R1, Female, District Director of Health Services)

The general view of respondents was that contrary to reports and policy documents that presented PHC as a community-driven programme with decisions and actions flowing gradually from the bottom to the top based on local needs, PHC implementation decisions and actions in practice were made from the top and gradually transferred downwards:

‘… [*T*]op-down. Policies are done centrally in Accra and brought here to implement. Bottom-up is just on paper. The operational manuals and guidelines we are using here were made by some people somewhere in Accra or in some donor country.’ (R9, Male, District Director of Health Services)

Respondents were however divided on the merits and appropriateness of the top-down approach to PHC implementation in Ghana. Proponents of the approach believed it was effective in reducing corruption, ensuring standardisation, uniform development and effective supervision of PHC interventions across the country. Opponents on the other hand believed it limited the opportunities available to PHC beneficiaries in policymaking and implementation, fostered apathy and disempowered local people and deprived them of opportunities to make genuine contributions to PHC’s development in their local area:

‘The top-down approach fosters apathy because the locals who are supposed to benefit are not involved or informed about PHC decisions.’ (R12, Female, District Director of Health Services)

Whilst PHC appeared to be more associated with rural, poor and underserved communities, findings also showed its growing importance in semi-urban, cosmopolitan and overpopulated settlements, especially in the areas of water and sanitation, and the fight against communicable diseases. Findings further suggest that more emphasis was placed on medical-oriented and donor-supported PHC initiatives than others. Similarly, PHC implementation activities were not uniform across the country, with marked differences existing in PHC activities and outcomes across regions, districts and communities:

‘We prioritise certain conditions depending on the disease profile of individual districts.’ (R19, Male, District Director of Health Services)

Although all respondents acknowledged that ideally PHC, per the Alma Ata declaration and per official GHS policies, was comprehensive in nature, PHC was in practice approached more as a selective, controlled and programme-based activity:

‘The official stance is comprehensive, but as you can see here, all we have are control programmes on targeted health priorities with different approaches and goals.’ (R17, Male, Deputy Regional Director of Health Services)

Furthermore, consensus amongst respondents was that the selective implementation of PHC was the result of the absence of resources, systems, structures, political will and international community support for comprehensive PHC in Ghana after Alma Ata. Consequently, key stakeholders had resorted to selective PHC as a low-cost yet efficient way of prioritising the Alma Ata goals consistent with the existing structures:

‘Comprehensive PHC was just beyond our resource capacity so we have always worked with the selective option.’ (R11, Female, Regional Health Research Officer)

Respondents’ views also suggest that the selective implementation of PHC had resulted in the duplication of efforts, uneven PHC outcomes across the country, limited local participation and affected the sustainability of vertical programmes, especially in the absence of donors. It further challenged the attainment of the PHC goals and required much more effort in monitoring and synergising the individual interventions, especially when they were managed by different stakeholders:

‘… [*I*]t is very difficult to manage the many concurrently running control programmes.’ (R15, Female, Director of Health Promotion Division)

In spite of its challenges, respondents generally believed that selective PHC was more suitable for Ghana’s unique PHC environment than the comprehensive approach. The common view was that it was cheaper and allowed for the concentration of scarce resources on particular health problems for maximum impact. In addition, it provided an opportunity for multiple stakeholders to select and make targeted contributions to particular PHC conditions of their choice:

‘Comprehensive is ideal but it is a resource dense activity which we can’t afford.’ (R18, Male, Regional Director of Health Services)

## Discussion

Whilst arguments about the concept of PHC remain theoretical, Magawa^31^ draws a strong link between actual PHC practice and perceived meanings held by implementers such that implementation activities were likely to be influenced by the perspectives of key implementers rather than official policy directions. Consequently, the varying meanings given to PHC in Ghana by its key implementers may therefore account for the varying manifestations of PHC activities in various parts of the country. Furthermore, findings support the arguments of Hogg et al.^32^ on the ambiguity associated with the PHC definition and the challenge of getting a universally applicable meaning of PHC.

In contrast to findings that key persons in charge of PHC implementation had little knowledge on the components of PHC and how they contributed to the attainment of the HFA2000,^33^ respondents were very knowledgeable and aware of the components and elements of PHC. However, their knowledge levels varied depending on their level in management and responsibility such that persons at the national levels appeared more knowledgeable than those at the district levels. This supports the view of May^34^ that knowledge and awareness of policy activities were dependent on how high and involved policy actors were in policy formulation.

Although the Alma-Ata Summit explicitly prescribed a bottom-up approach,^31^ PHC has been approached differently by different countries.^35^ In developing countries where public service provision is generally not decentralised for instance, the top-down approach is common.^36^ This appeared to be the case in Ghana, with respondents showing how the centralised structure and control of vertical programmes and health services delivery in general had cemented the top-down implementation of PHC in practice. This is in spite of GHS reports and policy statements suggesting the opposite. Findings, in this respect, provide valuable insights into the dichotomous relationship between PHC policy pronouncements and actual implementation practices, and further contribute by identifying key factors underpinning this discrepancy. Specifically, this study supports the view of Alesch et al.^37^ that weak institutional and resource capacity in local communities and the need for uniformity in policy implementation and outcomes were important reasons for the top-down implementation of PHC.

The top-down, bottom-up debate is not merely about the flow of policy decisions but a reflection of power relationship, control of resources and outcomes, prioritisation of initiatives and the determination of relative importance of methods used.^38,39^ Whilst bottom-up approaches empower local people to take control of their health,^40^ top-down approaches strengthen the control of central agencies on PHC.^41^ Narratives from respondents however show that the continual use of top-down approaches disempower communities, increase their dependency on external support and limit commitment and ownership of interventions. On a positive note, however, respondents associated top-down approaches with a reduction in corruption, standardisation, uniform development and effective supervision of PHC interventions across the country.

Clearly, both approaches appear to have various merits depending on the context, nature of policy and stakeholder preferences in implementation such that no single approach may in practice be suitable for all interventions or for all contexts. Consequently, prescribed approaches during the policy formulation stage may vary from those actually used in implementation.^36^ This was exemplified by the meanings constructed by PHC managers in this study that showed a practice of top-down implementation in contrast to Alma Ata’s bottom-up recommendation. Whilst similar findings have been made by Collins and Green,^42^ this study is distinctive in linking the existing structure and implementation systems of institutions tasked with implementation of the actual approach used in implementation. In Ghana’s case, the top-down architecture of the GHS, MoH and other key institutions tasked with PHC implementation was identified by respondents as a key factor behind the top-down implementation approaches used in PHC. Generally, top-down approaches regardless of their merits tend to sideline beneficiaries in implementation decisions.^43,44^ Similarly, respondents believed that top-down systems deprived PHC beneficiaries the chance to meaningfully contribute to PHC decisions that affect their health and everyday lives.

Universally, PHC implementation approaches and their respective outcomes have been strongly influenced by the comprehensive versus selective debate even amongst researchers and practitioners in the same context.^11^ Findings in this regard show the lack of consensus amongst respondents on whether PHC was practised comprehensively or selectively in Ghana. In support of Magnussen et al.,^45^ findings further suggest that Ghana like other developing countries recognise and present PHC policy as a comprehensive attempt to operationalise the Alma Ata goals. In practice, however, PHC implementation has gradually transitioned, either as a planned strategy or, by default, from its comprehensive intent to a more selective practice.^46,47^ The former was a key feature of interventions in Latin America where attention was given to individual components of PHC on an incremental basis within a defined period of time.^48^ Ghana’s case according to the findings reflects the latter where, under pressure from the environment and PHC financiers, selective PHC became the default implementation strategy. Similar findings on how selective PHC gained grounds under the auspices of key PHC financiers in developing countries like the United Nations Children’s Fund (UNICEF) and World Health Organization (WHO) have been made by Newell^47^ and Cueto.^49^

A close examination of the conceptual underpinnings of selective PHC in the face of study findings allows a deconstruction of some key misconceptions about its practice and implications in Ghana. In the first instance and contrary to some respondents views, Selective Primary Health Care (SPHC) was presented by Walsh and Warren^50^ as an interim strategy for disease control and not a direct replacement of the comprehensive approach. Nor was it designed to provide a long-term implementation scheme for HFA2000.^47^ Stakeholder perspectives however suggest that lack of resources, donor influence and the absence of the political will to restructure the existing health system has made SPHC a permanent strategy for PHC interventions with associated limitations on its usefulness in ensuring long-term PHC success. Theoretically, its use was also to be restricted to disease control elements in PHC in low-income countries that were not fully resourced to implement the full extent of the comprehensive approach.^51^ Within this context, participants believed that the general application of the disease-control-oriented selective PHC had furthered the medicalisation of PHC by emphasising immunisation, treatment of diseases and other medical-oriented PHC activities at the expense of Health Promotion(HP). Finally, SPHC was strongly presented and supported by UNICEF as a cost-effective way of achieving child health goals of PHC.^52^ Whilst it may therefore suffice for child health interventions, Macdonald^12^ believes it may be less suitable for other PHC initiatives. Respondents in confirmation explained how the Growth monitoring, oral rehydration, breast-feeding and immunisation (GOBI) and GOBIFFF SPHC had accelerated maternal and child health outcomes in Ghana but had been of little use in promoting water, sanitation and the provision of essential drugs. In relation, stakeholder views on the associated costs of managing several vertical programmes often with divergent goals simultaneously support Andrews and Crooks^53^ views that cost-effectiveness of SPHC does not necessarily make it the cheapest option for attaining global health targets.

Respondents’ views on the limitations in the attainment of overall PHC targets as a result of disparate outcomes in individual PHC components support Magnussen et al.^45^ that implementation must be done in a systemic manner bearing in mind that the ultimate improvement in health and inequality depends not only on the relative success of the individual elements but also on the collective success of all interventions. Findings further confirm LaFond’ views^54^ that PHC, as a system, has far-reaching interrelating and interdepending components within a dynamic policy environment, which must be implemented comprehensively. Thus, whilst selective approaches to PHC may improve selected dimensions of health, or may control the incidence and prevalence of particular public health cases, a holistic approach provides the easiest means of ensuring a more balanced improvement in the community’s health. In addition, respondents linked SPHC to a reduced sense of awareness and commitment to tackling the social determinants of health in developing countries.

Although PHC is implemented as a national programme in Ghana, findings show marked inequalities in the geographical distribution and utilisation of PHC services consistent with Getu and Devereux.^55^ In line with Logie et al.,^56^ findings show that PHC appears to be more evident in rural settlements and poor communities across the country although it had significant relevance to peri-urban and cosmopolitan areas plagued with the double burden of disease. Practically, it supports Quashigah’s views^57^ that PHC initiatives like those on basic sanitation and safe water present the surest means of tackling epidemics, effects of sedentary living and other health conditions associated with overpopulated urban areas in Ghana. Yet, and in line with Quashigah,^58^ respondents’ narratives show how its continual association with deprived and resource-constrained settlements lacking medical personnel and facilities have also limited its relevance and patronage in middle- and upper-income zones.

## Conclusion

In general, this study supports earlier studies that the implementation of PHC since its inception in developing countries has been lethargic, inconsistent and marred by controversies in terms of the right approach to its implementation. Specifically, however, this study shows that the nature and scope of PHC implementation in Ghana is premised by the lack of clarity on its meaning, components and limits. Within this context, PHC in Ghana and other sub-Saharan countries has generally metamorphosed from its comprehensive, local community-driven, bottom-up and people-centred approach to a medically oriented, donor-driven, selective and predominantly top-down activity. An examination of the approach to PHC implementation by this study therefore contributes to efforts at sustaining PHC and keeping its relevance within the Sustainable Development Goals (SDGs) by understanding how meanings and contextual issues of implementation can influence the direction and outcomes of global health initiatives. The recurring theme of a medicalised PHC system, for instance, had a strong effect on implementation with more attention being given to medical personnel, systems and interventions within PHC than health promotion and other non-medicine-based activities. Similarly, vertical programmes under the selective PHC framework also limited community participation and empowerment whilst increasing dependence of local communities on the health system and development partners and donors.

Importantly, this study provides evidence of the existence of gaps between policy and practice in several spheres of PHC activities and management in Ghana and other developing countries. Constant references to distinctions between what happened on paper or in policy documents and what actually happened on the ground or during implementation, for instance, suggest a dichotomous relationship between policy formulation and implementation with respect to PHC and the gatekeepers that manage it in Ghana. Whilst throwing light on the wide-reaching context-specific factors that inhibit the translation of policy objectives into actionable outcomes, the broad acknowledgement and acceptance of these variances between policy and practice casts noteworthy doubts on the veracity of reports, reviews and other official evaluations on PHC.

Finally, this study draws attention to how donors and external development partners have exerted strong control over PHC decisions and actions through funding, support and prioritisation of particular PHC interventions and control programmes. In many instances and contrary to the PHC narrative of community participation, the preferences of these stakeholders during implementation superseded the preferences of local communities and the management responsibility of the GHS and MoH. Consequently, this study recommends further studies on the resource constraints and role of external stakeholders in PHC implementation to provide more insights into the subject as well as to augment the current discourse on sustaining PHC in developing countries. Considering the relatively small sample and its limited application to other developing countries, this study recommends further research in other countries on context-specific issues affecting PHC implementation and implementers.
